# A Pro-Inflammatory Gut Microbiome Characterizes SARS-CoV-2 Infected Patients and a Reduction in the Connectivity of an Anti-Inflammatory Bacterial Network Associates With Severe COVID-19

**DOI:** 10.3389/fcimb.2021.747816

**Published:** 2021-11-17

**Authors:** Johanna Reinold, Farnoush Farahpour, Christian Fehring, Sebastian Dolff, Margarethe Konik, Johannes Korth, Lukas van Baal, Daniel Hoffmann, Jan Buer, Oliver Witzke, Astrid M. Westendorf, Jan Kehrmann

**Affiliations:** ^1^ Department of Infectious Diseases, University Hospital Essen, University of Duisburg-Essen, Essen, Germany; ^2^ Bioinformatics and Computational Biophysics, University Duisburg-Essen, Essen, Germany; ^3^ Institute of Medical Microbiology, University Hospital Essen, University of Duisburg-Essen, Essen, Germany; ^4^ Department of Nephrology, University Hospital Essen, University of Duisburg-Essen, Essen, Germany; ^5^ Department of Endocrinology, Diabetes and Metabolism, University Hospital Essen, University Duisburg-Essen, Essen, Germany

**Keywords:** COVID-19, microbiome, intestinal microbiota, SARS-CoV-2, severity

## Abstract

The gut microbiota contributes to maintaining human health and regulating immune responses. Severe COVID-19 illness is associated with a dysregulated pro-inflammatory immune response. The effect of SARS-CoV-2 on altering the gut microbiome and the relevance of the gut microbiome on COVID-19 severity needs to be clarified. In this prospective study, we analyzed the gut microbiome of 212 patients of a tertiary care hospital (117 patients infected with SARS-CoV-2 and 95 SARS-CoV-2 negative patients) using *16S rRNA* gene sequencing of the V3-V4 region. Inflammatory markers and immune cells were quantified from blood. The gut microbiome in SARS-CoV-2 infected patients was characterized by a lower bacterial richness and distinct differences in the gut microbiome composition, including an enrichment of the phyla Proteobacteria and Bacteroidetes and a decrease of Actinobacteria compared to SARS-CoV-2 negative patients. The relative abundance of several genera including *Bifidobacterium*, *Streptococcus* and *Collinsella* was lower in SARS-CoV-2 positive patients while the abundance of *Bacteroides* and *Enterobacteriaceae* was increased. Higher pro-inflammatory blood markers and a lower CD8^+^ T cell number characterized patients with severe COVID-19 illness. The gut microbiome of patients with severe/critical COVID-19 exhibited a lower abundance of butyrate-producing genera *Faecalibacterium* and *Roseburia* and a reduction in the connectivity of a distinct network of anti-inflammatory genera that was observed in patients with mild COVID-19 illness and in SARS-CoV-2 negative patients. Dysbiosis of the gut microbiome associated with a pro-inflammatory signature may contribute to the hyperinflammatory immune response characterizing severe COVID-19 illness.

## Introduction

Since December 2019, coronavirus disease 2019 (COVID-19) caused by severe acute respiratory syndrome coronavirus type 2 (SARS-CoV-2) has rapidly spread globally and evolved to the most severe pandemic of the last 100 years with more than 191.1 million confirmed infected individuals and 4.1 million deaths since December 2019 (accessed 22.7.2021) (WHO, 2020). Although SARS-CoV-2 primarily infects the lungs and fever, cough and dyspnea are the most common clinical manifestations (Guan et al., 2020). COVID-19 is recognized as a multi-organ disease associated with hepatic, nephrological, neurologic, cardiac, and gastrointestinal complications.

Clinical and experimental studies reveal a fundamental role of the gut microbiota for human health (Negi et al., 2019; Zheng et al., 2020). Microbiota-host interactions regulate immune responses in distant organs by microbial metabolites, microbial associated molecular patterns and interactions between microorganisms and immune cells. The gut microbiota influences the outcome of infectious lung diseases in animal models (Schuijt et al., 2016; Steed et al., 2017) and *vice versa*, pulmonary infections affect the gut microbiota (Wang et al., 2014; Yildiz et al., 2018), implying a bidirectional cross-talk between the gut and the lungs (Sencio et al., 2021). SARS-CoV-2 RNA is detected in stool samples in about 50% of infected patients, even when the virus is no longer detected in the respiratory tract (Xiao et al., 2020). Diarrhea as a common extrapulmonary symptom and the detection of infectious SARS-CoV-2 in stool samples suggest that the gastrointestinal tract might be a site for active virus replication and that the virus might directly disturb the local ecosystem in the gut (Gu et al., 2020a; Parasa et al., 2020). The replication of SARS-CoV-2 in enterocytes using small intestinal organoids and the high expression of cell surface receptor angiotensine-converting enzyme II (ACE2) by enterocytes, support the hypothesis of active replication in the gut and disturbance of the gut microbiome (Hashimoto et al., 2012; Xiao et al., 2020). ACE2 expression is downregulated by interaction with coronaviruses and influences gut homeostasis by affecting tryptophan metabolism and production of antimicrobial peptides (Perlot and Penninger, 2013).

The severity of acute COVID-19 varies from asymptomatic infection to life-threatening acute respiratory distress syndrome. While the immune response in asymptomatic individuals or patients with mild disease is characterized by activation of CD8^+^ T cells and Th1 cells, a dysregulated hyperinflammatory syndrome characterizes the immune response in patients with severe or critical COVID-19 (Garcia, 2020; Brodin, 2021). Ethnicity and regional factors are major variables affecting the gut microbiome composition (Deschasaux et al., 2018; Ghosh et al., 2020) and studies investigating the gut microbiome in SARS-CoV-2-infected patients have been reported mainly from Asian populations in cohorts with a significant proportion of patients receiving antibiotic treatment. In addition, high age, obesity (Simonnet et al., 2020), and diabetes (Gao et al., 2020) are risk factors associated with the severe course of disease which have been linked to alterations of the gut microbiome previously.

This prospective study analyzed the gut microbiome using *16S rRNA* gene sequencing in 212 patients with 98% Caucasian ethnicity including 117 SARS-CoV-2 positive and 95 SARS-CoV-2 negative patients of a German tertiary care hospital. SARS-CoV-2 positive and negative patients did not differ for various variables associated with alterations of the gut microbiome and only 3% of patients were treated with antibiotics at the time point of sampling for both groups. The periods between first symptoms of COVID-19 and rectal swab sampling did not differ significantly between patients of different COVID-19 severity categories. We analyzed the gut microbiome differences linked to SARS-CoV-2 infection and those according to COVID-19 severity illness from rectal swab samples.

## Material and Methods

### Study Population

This study was reviewed and approved by the Ethics Committee of the Medical Faculty of the University of Duisburg-Essen (20-9237-BO) and was performed in accordance with the latest version of the Declaration of Helsinki. Written informed consent was obtained from all patients before enrolment. A total of 212 patients presenting in the tertiary care University Hospital Essen were included between April and November 2020, including 117 SARS-CoV-2 positive and 95 SARS-CoV-2 negative patients with other reasons for hospital presentation. SARS-CoV-2 real-time PCR from nasopharyngeal swabs was used for detection of acute SARS-CoV-2 infection in all patients enrolled in this study. Data collection included anamnesis, clinical examination and letters of the patient’s medical history. Pneumonia was diagnosed by low dose computer tomography of the lungs and was performed in 79 patients with moderate respiratory symptoms. Alternatively, chest x-ray was performed in SARS-CoV-2 positive patients. COVID-19 severity illness was classified according to the WHO (Rochwerg et al., 2020). Mild disease was defined as SARS-CoV-2 infection without pneumonia, moderate COVID-19 was classified as pneumonia with a saturation of peripheral oxygen (SpO2) > 90% on room air and typical signs such as fever and cough. Severe COVID-19 was defined as symptomatic pneumonia with SpO2 < 90% on room air and a respiratory rate > 30 breaths per minute or clinical signs of severe dyspnoea. Critical disease was classified by the need of life sustaining treatment e.g. acute respiratory distress syndrome or septic shock. WHO unites mild and moderate disease as non-severe COVID-19. For microbiome analyses assessing differences associated with disease severity, severe and critical COVID-19 patients were assigned to one category (severe/critical) because of the low number of patients with critical COVID-19 (n=12).

Comprehensive blood tests assessing functions of multiple organs including kidney, liver, and bone marrow, as well as blood count and C-reactive protein were available for 113 SARS-CoV-2 positive patients from the same day the rectal swab was taken. Additional parameters describing inflammatory response and immune cell characteristics including IL6, sIL-2R and numbers of CD3^+^ T cells, CD19^+^ B cells, CD4^+^ T cells, CD8^+^ T cells, HLA-DR^+^ T cells, CD3^-^CD16^+^CD56^+^ NK cells, and CD3^+^CD16^+^CD56^+^ NK T cells were quantified by flow-cytometry in 50 patients 1.5 days after rectal swab sampling.

### Sample Processing

Rectal swab samples were taken using the DNA/RNA Shield™ Collection Tube with Swab (Zymo Research, Freiburg, Germany) and stored in stabilizing DNA/RNA shield at -80°C until DNA extraction was performed. DNA was extracted using the ZymoBIOMICS DNA Miniprep Kit (Zymo Research) with a bead-beating step using the Fast-Prep device (MP Biomedicals, Santa Ana, CA) before DNA extraction was performed. Library preparation was performed using the NEBNext^®^ Ultra II DNA Library Prep Kit. The V3/V4 hypervariable region of the bacterial 16S rRNA gene was amplified with 341F (5′-CCTAYGGGRBGCASCAG-3′) and 806R (5′-GGACTACNNGGGTATCTAAT-3′) primers and samples were sequenced on NovaSeq 6000 SP flowcell with PE250. DNA stabilizer liquid fluid from the collection tubes undergoing the DNA-extraction procedure and a PCR water sample were used as negative controls.

### Microbiome Data Analysis

We analyzed demultiplexed fastq-files of forward and reverse reads per sample using the QIIME2 (Quantitative Insights Into Microbial Ecology2) pipeline (Bolyen et al., 2019). Sequences were corrected and quality filtered using the DADA2-package integrated in QIIME2 including filtering of chimeric sequences using the consensus-method. We obtained a total of 16,915,240 quality-filtered sequences of 212 samples with a minimum of 29,676 sequences, maximum of 117,553 sequences per sample and a mean/median number of 79,789/84,742 sequences per sample. We used the q2 diversity plugin for performing different alpha diversity and beta diversity metrics with a rarefaction depth of 29,676 sequences, which represents the sample with the minimum number of sequences. The q2-feature-classifier plugin was used for assigning taxonomy with a Naïve Bayes classifier, trained on the Greengenes 13_8_99% OTUs *16S rRNA* gene full length sequences. Alpha diversity metrics, barplots, boxplots and principle coordinates analysis (PCoA) were visualized using Dokdo, Version 1.7. Comparisons between categorical metadata columns and alpha diversity metrics were computed using the *qiime diversity alpha-group-significance* command with Kruskal-Wallis test. To test for significant differences in beta diversity among groups, we used the *qiime diversity beta-group significance* command with permutational multivariate analysis of variance (PERMANOVA) of distance matrices with 999 permutations. For analysis of differences in the relative taxa abundance between groups, taxa with a relative abundance of at least 0.001% of the total reads were filtered and Kruskal–Wallis test was used for analysis of significant differences between groups using Calypso (Zakrzewski et al., 2017). Benjamini–Hochberg false discovery rate (FDR) correction for multiple hypothesis testing was performed when indicated. Biomarkers were assessed using LEfSe (Linear discriminant effect size analysis) (Segata et al., 2011) with a P<.05 for bacterial class comparison using Kruskal-Wallis test and a linear discriminant analysis (LDA) score >3.5.

The co-occurrence analysis was performed as described previously (Kehrmann et al., 2019). The analysis was done at the genus level. To remove the effect of unbalanced sample sizes in different groups, the minimum acceptable threshold was calculated by iterative sub-sampling inside the groups with the smallest group size (N=35). Co-occurrence networks were visualized in Cytoscape, version 3.7.1. Graph layout was obtained by applying multidimensional scaling on the co-occurrence matrix of the negative group.

Patient characteristics were tested using t-test for metric variables and Fisher exact test for categorical variables with a significance level <.05 using SPSS software (version 27.0).

## Results

### Clinical Characteristics of the Study Population

The study population included 212 patients in total. Of 117 SARS-CoV-2 positive patients, only 4 (3%) were treated with antibiotics before rectal swab sampling, which was comparable to the number of antibiotics-treated patients that were SARS-CoV-2 negative (3 of 95, 3%) (**Table 1**). Variables that have been associated with alterations of the gut microbiome including ethnicity, sex, body-mass-index (BMI) and smoking status as well as medication taken at the time point of sampling including metformin, proton-pump inhibitors, and corticosteroids did not differ significantly between SARS-CoV-2 positive and negative patients. The period between admission to hospital and time point of rectal swab sampling was comparable for both groups as well as the percentage of patients that died in hospital. SARS-CoV-2 positive patients were characterized by slightly higher mean age (56.2 years *vs* 50.2 years, p=.033), longer hospital stay with a mean of 9 days *vs.* 4 days (p=.01) and by a higher percentage of patients that were admitted to intensive-care unit during hospitalization (p=.025). Comorbidities did not differ between both groups. The symptoms cough, fever, gastrointestinal symptoms, diarrhea and odor disorders were more prevalent in SARS-CoV-2 infected patients.

**Table 1:** Patient characteristics of SARS-CoV-2 positive and negative patients.

Variables	SARS-CoV-2 positive	SARS-CoV-2 negative	P-value
Number of subjects	117	95	
Age, years, mean ± SD	56 ± 19	50 ± 21	.033
Sex, male	57 (49)	49 (52)	.782
Body mass index, kg/m², mean ± SD	28 ± 6	29 ± 8	.484
Smoker	24 (22)	31 (34)	.83
**Outcome & Complications**			
Length of hospitalization	9 ± 9	5 ± 6	<.001
Time between hospitalization & rectal swab, days, mean ± SD	2 ± 3	1 ± 3	.263
Admission to intensive care unit	10 (9)	1 (1)	.014
Deceased	4 (3)	3 (3)	1.0
**Specific symptoms**			
Diarrhea	37 (32)	6 (6)	<.001
Odor disorder	32 (29)	0	<.001
Fever	71 (61)	8 (8)	<.001
Cough	82 (71)	13 (14)	<.001
**Comorbidities**			
Pre-existing illness	89 (76)	67 (76)	1.0
Diabetes mellitus	24 (21)	28 (30)	.148
Coronary heart disease	16 (14)	18 (19)	.348
Hypertension	43 (37)	41 (46)	.199
Chronic kidney disease	9 (8)	16 (17)	.052
Solid organ transplantation	3 (3)	3 (3)	1.0
Chronic obstructive pulmonary disease	15 (13)	16 (17)	.439
Malignancy	18 (15)	15 (16)	1.0
**Medication at the timepoint of sampling**			
Proton pump inhibitor	39 (35)	28 (32)	.763
Metformin	6 (5)	5 (5)	1.0
Laxatives	4 (3)	0	.131
ACE inhibitor	38 (33)	39 (44)	.110
Beta blocker	37 (32)	35 (38)	.383
Platelet aggregation inhibitor	21 (18)	18 (19)	.859
Statin	22 (19)	20 (22)	.729
Steroid	12 (10)	16 (17)	.159
Remdesivir	3 (3)	0	1.0
Antibiotics	4 (3)	3 (3)	1.0

Data are presented as numbers (%) unless otherwise indicated. Metric variables were tested using t-test with mean ± SD. Fisher’s exact test was used for categorical variables. ACE, angiotensin-converting enzyme; SD, standard deviation.

### Differences in the Gut Microbiome Linked to SARS-CoV-2 Infection

**Figure 1:**
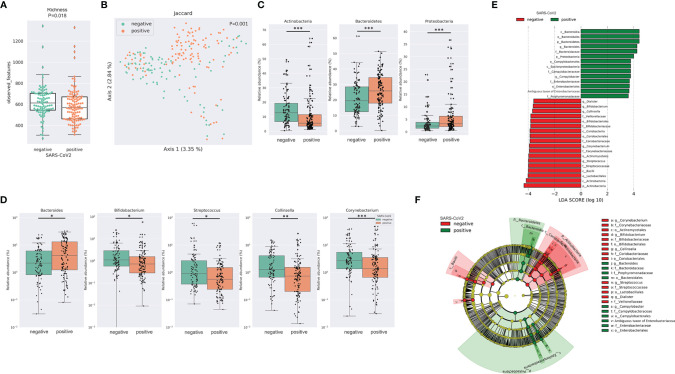
SARS-CoV-2 infected patients exhibit distinct differences in the gut microbiome **(A)** Bacterial richness in SARS-CoV-2 positive and negative patients determined by observed features (amplicon sequence variants [ASV]). Kruskal-Wallis test was used to test for significant differences among groups. * FDR-P < .05; ** FDR-P < .01¸*** FDR-*P < .001* **(B)** Principal Coordinates Analysis (PCoA) of SARS-CoV-2 positive performed with of Binary Jaccard distance matrix. PERMANOVA multivariate analysis was used to test for significant differences. **(C)** Differences in the relative abundance of bacterial phyla linked to SARS-CoV-2 infection. **(D)** Illustration of the differences in the relative abundance of bacterial genera linked to SARS-CoV-2 infection. **(E)** Linear discriminant effect size (LEfSe) analysis identified several bacterial taxa enriched in SARS-CoV-2 positive (green) and SARS-CoV-2 negative patients (red) **(F)** Cladogram reports the taxa showing different abundance values (LDA>3.5) according to LEfSe. Colors are presented in the color of the most abundant group (SARS-CoV-2 positive in green, SARS-CoV-2 negative in red).

After merging and quality filtering of sequencing reads, we received a total of 16,915,240 sequences for 212 patient samples with a mean of 79,789, a minimum of 29,676 and a maximum of 117,553 sequences per sample. The bacterial richness was significantly lower in SARS-CoV-2 positive compared to SARS-CoV-2 negative patients (**Figure 1A**) while Shannon diversity index and Pielou’s evenness were not significantly different between the two groups (**Supplemental Figure 1A**). Loss of microbial richness of the gut microbiome is associated with chronic diseases and a pronounced inflammatory phenotype (Le Chatelier et al., 2013). Principal Coordinates Analysis and PERMANOVA multivariate analysis of distance matrices of Binary Jaccard, Bray-Curtis and weighted and unweighted UniFrac revealed differences between SARS-CoV-2 positive and negative patients (p=.001 for multivariate analyses of all four distance matrices, **Figure 1B** and **Supplemental Figure 1B**). Interestingly, the relative abundance of three of the four dominating bacterial phyla was significantly different between the two groups. While the relative abundance of Proteobacteria and Bacteroidetes was significantly higher in SARS-CoV-2 positive patients, the relative abundance of the phylum Actinobacteria was lower in these patients (**Figure 1C**). Of the 20 most abundant genera, 5 were significantly different between SARS-CoV-2 positive and negative patients. While the relative abundance of *Bacteroides* was higher in SARS-CoV-2 positive patients, the relative abundance of *Bifidobacterium*, *Collinsella*, *Streptococcus* and *Corynebacterium* were higher in SARS-CoV-2 negative patients (**Figure 1D**). To further evaluate differences in the gut microbiome between both groups, we performed class comparisons using linear discriminant analysis of effect size (LEfSe) that identified several bacterial taxa that discriminate SARS-CoV-2 positive from SARS-CoV-2 negative patients with LDA score >3.5. LEfSe confirmed enrichment of Proteobacteria and Bacteroidetes phyla and Bacteroides genus in SARS-CoV-2 positive patients and additionally identified a higher relative abundance of pro-inflammatory *Enterobacteriaceae* and *Campylobacteraceae* families as a biomarker for SARS-CoV-2 positive patients, while Actinobacteria phylum and genera *Bifidobacterium*, *Collinsella*, *Streptococcus*, *Corynebacterium*, and orders *Lactobacillales*, *Actinomycetales* among others were identified as biomarkers for SARS-CoV-2 negative patients (**Figures 1E, F**).

The differences in the gut microbiome between SARS-CoV-2 positive patients with and without diarrhea for alpha and beta diversity metrics were minor (**Supplemental Figure 2**), and LEfSe did not identify any taxon with LDA >3.5 that discriminated patients with and without diarrhea.

### Differences in the Gut Microbiome Linked to COVID-19 Severity Illness

**Figure 2:**
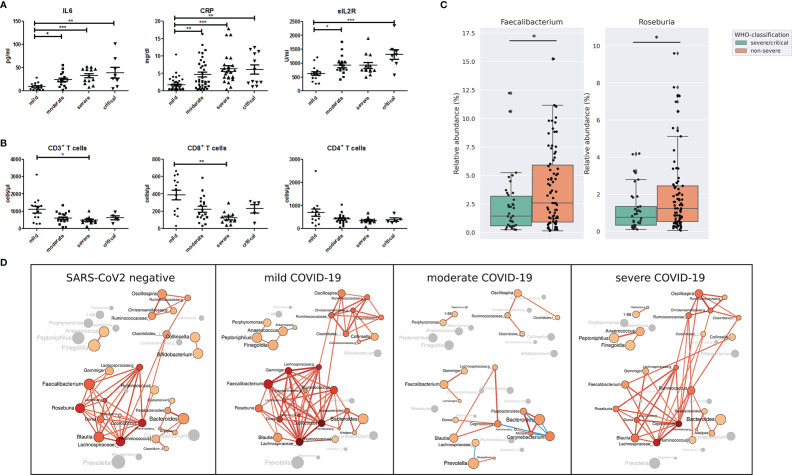
**(A)** Serum levels of pro-inflammatory markers interleukin 6 (IL-6), C-reactive protein (CRP) and soluble IL-2 receptor (sIL2R) and **(B)** total number of CD3^+^ T cells, CD8^+^ T cells and CD4^+^ T cells in COVID-19 patients according to disease severity. *P <.05; **P < .01¸***P < .001 **(C)** Relative abundance of bacterial genera *Faecalibacterium* and *Roseburia* in patients with non-severe and severe/critical COVID-19 according to the WHO classification. *P < .05 **(D)** Gut microbiota co-occurrence networks are visualized for SARS-CoV-2 negative patients, patients with mild, moderate and severe COVID-19 severity illness. Nodes represent bacterial genera. The size of each node indicates the average relative abundance of the genus in the corresponding group and each node is colored based on the degree of the node (number of edges). Labels are scaled with the size of the nodes. The intensity of the red color of the nodes increases according to the number of the genera’s edges. Genera without a significant co-occurrence are colored in gray in the group that lacks a co-occurrence partner. The width of the edges are weighted based on the absolute value of the co-occurrences and colored red for positive and blue for negative correlations.

According to the World Health Organization classification (WHO) COVID-19 classification, 26 patients of our study were classified as severe COVID-19 with radiological signs of pneumonia, SpO2<90% on room air, respiratory rate >30, and signs of severe respiratory distress. Twelve patients were classified as critical COVID-19 requiring life-sustaining treatment, acute respiratory distress syndrome and sepsis or septic shock, while 79 patients were classified as non-severe COVID-19 classified by the absence of signs of severe and critical COVID-19 as defined by the WHO (**Table 2**). Age (P <.001), BMI (p=.053) coronary heart disease (p=.02), hypertension (p=.001), chronic kidney disease (p=.008) as well as ACE-inhibitor, beta blocker (p=.0025) platelet aggregation inhibitor (p=.005), and Remdesivir medication (p=.041) were the variables that were significantly different between the groups according to disease severity. Other variables including diarrhea, diabetes mellitus, smoking status, use of proton pump inhibitors, the period between the first COVID-19 symptoms and gut microbiome sampling and the period between admission to hospital and rectal sampling were not significantly different between the groups according to COVID-19 severity (**Table 2**). Patients with severe and critical COVID-19 were characterized by higher levels of IL6, C-reactive protein and soluble IL2-receptor expression (**Figure 2A**). The total number of T cells was lower in patients with severe COVID-19 compared to those with mild COVID-19, driven by the lower number of CD8^+^ T cells. The CD4^+^ T cell number (**Figure 2B**) as well as the total numbers of B cells (CD19^+^), NK cells (CD3^-^CD16^+^CD56^+^), natural-killer like T cells (CD3^+^CD16^+^CD56^+^) and HLA DR^+^ T cells were not significantly different between the groups (**Supplemental Figure 3**).

**Table 2:** Patient characteristics of SARS-CoV-2 positive patients according to COVID-19 disease severity.

Variables	Mild	Moderate	Severe/critical	P value
Number of subjects	44	35	38	
Age, years, mean ± SD	47 ± 19	56 ± 17	67 ± 16	<.001
Male	21 (48)	16 (46)	20 (53)	.828
Body mass index, kg/m², mean ± SD	27 ± 5	28 ± 7	30 ± 6	.053
Smoker	12 (31)	5 (15)	7 (21)	.248
**Outcome & Complications**				
Length of hospitalization, days, mean ± SD	6 ± 7	7 ± 6	14 ± 10	<.001
Time between hospitalization & rectal swab, days, mean ± SD	2 ± 5	1 ± 3	1 ± 2	.263
Time between first symptoms & rectal swab, days, mean ± SD	8 ± 12	7 ± 7	6 ± 5	.827
Admission to intensive care unit	0	0	10 (27)	<.001
Deceased	1 (2)	0	3 (8)	.156
**Gastrointestinal symptoms**				
Diarrhea	10 (23)	14 (40)	13 (34)	.239
Odor disorder	10 (23)	13 (38)	9 (26)	31.318
**Comorbidities**				
Pre-existing illness	29 (66)	24 (69)	36 (95)	.004
Diabetes mellitus	7 (16)	6 (17)	11 (29)	.290
Coronary heart disease	4 (9)	2 (6)	10 (26)	.02
Hypertension	11 (25)	9 (26)	23 (61)	.001
Chronic kidney disease	2 (5)	0	7 (18)	.008
Solid organ transplantation	2 (5)	0	0	0.78
Chronic obstructive pulmonary disease	2 (5)	3 (9)	10 (26)	.009
Malignancy	5 (11)	7 (20)	6 (16)	.570
**Medication at time point of sampling**				
Proton pump inhibitor	14 (36)	10 (29)	15 (40)	.611
Metformin	4 (10)	0	2 (5)	.165
Laxative	2 (5)	2 (5)	0	.351
ACE inhibitor	7 (17)	10 (29)	21 (55)	.001
Beta blocker	8 (19)	11 (31)	18 (47)	.025
Platelet aggregation inhibitor	6 (14)	2 (6)	13 (34)	.005
Statin	8 (19)	4 (11)	10 (26)	.271
Steroid	6 (14)	2 (6)	4 (11)	.514
Remdesivir	0	0	3 (8)	.041
Antibiotics	1 (2)	0	3 (8)	.156

Data are presented as numbers (%) unless otherwise indicated. Metric variables were tested using t-test with mean ± SD. Fisher’s exact test was used for categorical variables. ACE, angiotensin-converting enzyme; SD, standard deviation.

Compared with the gut microbiome differences between SARS-CoV-2 positive and negative patients, the differences linked to COVID-19 severity were not as prominent. Nevertheless, the relative abundance of the genera *Faecalibacterium* and *Roseburia*, both important butyrate-producing bacterial genera, was lower in severe/critical COVID-19 compared with non-severe COVID-19 (**Figure 2C**). These two genera were the only ones that were identified by LEfSe to discriminate COVID-19 patients with severe/critical disease *versus* non-severe illness with LDA score >3.5. In addition, *Faecalibacterium* and *Roseburia* were among the central genera of the largest interconnected cluster of the co-occurrence network of SARS-CoV-2 negative patients and patients with mild COVID-19 severity illness (**Figure 2D**). This cluster also included the bacterial genera *Dorea*, *Blautia*, *Coprococcus*, *Lachnospira* and two genera of *Lachnospiraceae* family exhibiting multiple positive correlations among each other. These genera have anti-inflammatory properties and are producers of short-chain fatty acids (Louis and Flint, 2017). Interestingly, positive correlations of *Faecalibacterium* and *Roseburia* with most genera of this bacterial cluster detected in SARS-CoV-2 negative patients and patients with mild COVID-19 were not present in moderate and severe COVID-19.

## Discussion

**Figure 3:**
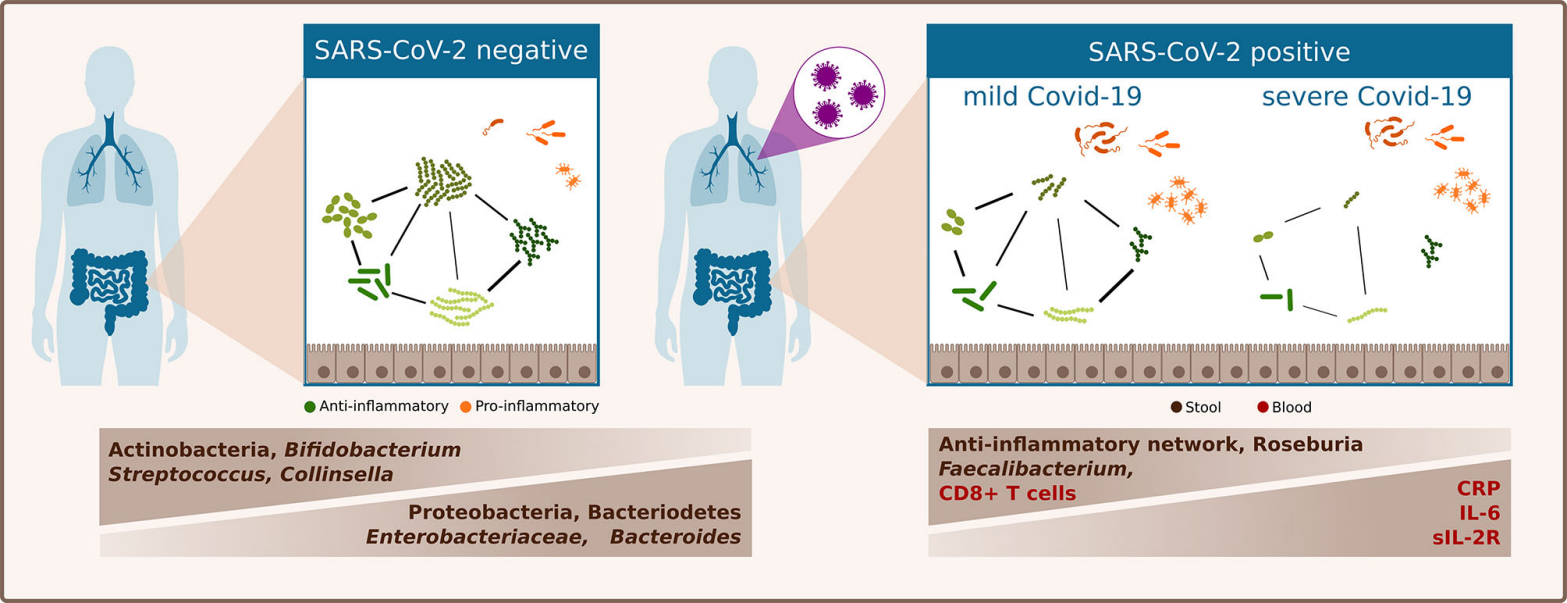
Schematic summary of differences of the gut microbiome linked to SARS-CoV-2 infection and COVID-19 severity illness.

In a European population cohort comprising mainly patients with Caucasian ethnicity, we showed that the gut microbiota in SARS-CoV-2 infected patients is clearly distinct from that of SARS-CoV-2 negative patients. Bacterial richness was lower in SARS-CoV-2 positive patients and the relative abundance of three of the four most abundant phyla differed between SARS-CoV-2 positive and negative patients. SARS-CoV-2 positive patients were characterized by a higher relative abundance of Bacteroidetes and Proteobacteria and a lower abundance of Actinobacteria. Furthermore, SARS-CoV-2 positive patients were characterized by an increase in *Bacteroides* genus, and *Enterobacteriaceae* family and decrease in *Bifidobacteria*. Patients with severe or critical COVID-19 exhibited an increased inflammatory immune response and their gut microbiome was characterized by a depletion of *Faecalibacterium* and *Roseburia* genera and by distinct alterations of bacterial networks (**Figure 3**).

A reduced bacterial richness of SARS-CoV-2 positive patients also characterizes the gut microbiome of chronic inflammatory and metabolic diseases and has been observed in viral respiratory infections (Le Chatelier et al., 2013; Yildiz et al., 2018). An increase of Proteobacteria of the human gut microbiome is a diagnostic signature for gut dysbiosis and risk of disease (Shin et al., 2015). The family *Enterobacteriaceae* of Proteobacteria phylum, enriched in SARS-CoV-2 positive patients of our study, has pro-inflammatory properties and is overrepresented in inflammatory diseases (Arumugam et al., 2011). The phylum Actinobacteria, depleted in SARS-CoV-2 positive patients of our study, plays a critical role in maintaining gut homeostasis and comprises the genus *Bifidobacterium*, which has beneficial effects for human health and was also depleted in SARS-CoV-2 positive patients (Binda et al., 2018). In a murine study, colonization of the gut microbiome with a *Bifidobacterium* species was shown to protect from severe influenza disease (Zhang et al., 2020).

COVID-19 severity illness is not only dependent on virus virulence but is also determined by the immune reaction. While the immune response in asymptomatic patients and patients with mild COVID-19 is characterized by infiltration of cytotoxic CD8^+^ T cells, IFN-driven Th1 response and production of neutralizing antibodies by plasma cells, an aberrant immune response with excessive inflammation, increased expression of pro-inflammatory mediators and cytokines characterizes severe and critical COVID-19 illness (Garcia, 2020; Brodin, 2021). In line with this, serum levels of pro-inflammatory markers IL-6, C-reactive protein (CRP) and soluble IL2-receptor (sIL-2R) expression were higher and number of cytotoxic CD8^+^ T cells was lower in patients with severe/critical COVID-19 compared to mild COVID-19 of our study.

Ethnicity, regional factors and socio-economic features are independent major factors affecting the gut microbiome (Brooks et al., 2018; Deschasaux et al., 2018). Regional factors had the highest effect on the gut microbiome composition and ranked before the variables age, gender, BMI and disease conditions in a dataset of 2,500 individuals investigating the gut microbiome composition of patients with inflammatory bowel disease (IBD), type2 diabetes, colorectal cancer, polyps and cirrhosis (Ghosh et al., 2020). Most of the studies investigating the differences in the gut microbiome between SARS-CoV-2 positive and negative patients have so far been published with data sets from Asian populations (Gu et al., 2020b; Tao et al., 2020; Zuo et al., 2020; Yeoh et al., 2021; Zuo et al., 2021). A reduced bacterial richness characterizes the gut microbiome of SARS-CoV-2 positive patients of our study, which is in line with two other studies (Gu et al., 2020b; Tao et al., 2020) assessing a reduced bacterial richness linked to SARS-CoV-2 infection using Chao1 index, while no differences were found by Yeoh et al. (2021). Shannon diversity was not significantly different between SARS-CoV-2 positive and negative patients of our study, which is in line with Yeoh et al. (2021), while Gu et al. reported a reduced Shannon diversity linked to COVID-19 (Gu et al., 2020b). Although alterations of the gut microbiota composition in patients with COVID-19 have been observed in the different studies, taxa associated with SARS-CoV-2 infection varied between studies. Nevertheless, an increase of bacteria with pathogenic and inflammatory properties and a decrease of beneficial anti-inflammatory bacteria were repeated findings. The enrichment of Bacteroidetes phylum and *Bacteroides* genus and lower abundance of Actinobacteria phylum and *Bifidobacterium* genus of our study is in line with a recent study from Asia that reported an enrichment in phylum Bacteroidetes and *Bacteroides dorei* in COVID-19 patients and a lower abundance of Actinobacteria and *Bifidobacterium adolescentis* (Yeoh et al., 2021). However, a decrease of Bacteroidetes phylum (Tao et al., 2020) or an increase of Actinobacteria (Gu et al., 2020b) was also reported. An enrichment of Proteobacteria phylum and *Enterobacteriaceae* family detected in SARS-CoV-2 positive patients of our study characterizes the gut microbiome in inflammatory diseases of the gut including IBD and obesity. An increase of defined taxa of the Proteobacteria phylum was a repeated finding in SARS-CoV-2 positive patients (Tao et al., 2020; Moreira-Rosário et al., 2021) or was associated with high faecal SARS-CoV-2 activity (Zuo et al., 2021).

ACE2, the receptor used for infection of host cells by SARS-CoV-2, is highly expressed in the human small intestine and involved in the production of antimicrobial peptides by regulating the tryptophan uptake into enterocytes (Hashimoto et al., 2012). The downregulation of ACE2 expression after interaction with coronaviruses might therefore influence the gut microbiota composition by inhibiting the production of antimicrobial peptides (Kuba et al., 2005). Studies correlating diarrhea and COVID-19 disease severity show conflicting results with reporting positive and negative correlations as well as no correlations as reviewed by Guo et al. (2021). In our cohort, diarrhea was not associated with disease severity. We did not aim to detect SARS-CoV-2 from rectal swabs in our study, but we compared the gut microbiome in COVID-19 patients with and without diarrhea and did not find major differences in COVID-19 patients linked to diarrhea.

Compared with the pronounced differences of the gut microbiome linked to SARS-CoV-2 infection, the differences associated with COVID-19 severity illness were modest. The period between first symptoms and rectal swab sampling was comparable for COVID-19 severity categories, indicating that differences between groups linked to COVID-19 severity are not explained by different phases of COVID-19. *Faecalibacterium*, which was represented by the species *Faecalibacterium prausnitzii*, and *Roseburia* were the only two bacterial genera in our study that discriminated patients with non-severe from those with severe/critical COVID-19. Both genera are associated with gut health and are important producers of the immune-modulatory short-chain fatty acid (SCFA) butyrate, which has anti-inflammatory properties and was shown to induce regulatory T cells which are indispensable for gut homeostasis (Furusawa et al., 2013; Machiels et al., 2014). The reduction of bacterial genera with anti-inflammatory properties and the reduction of the bacterial network consisting of genera with anti-inflammatory properties may contribute to the pro-inflammatory phenotype with excessive production of inflammatory cytokines and markers existing in patients with severe/critical COVID-19 of our study. To our knowledge, a reduction of the connectivity of an anti-inflammatory bacterial network of the gut microbiome described in our study has not been reported previously. SCFA from high-fiber diet protect mice from severe influenza infection by enhancing CD8^+^ T cell immunity (Trompette et al., 2018). Depletion of *Faecalibacterium prausnitzii* and *Roseburia* was observed in ulcerative colitis (Machiels et al., 2014) and also identified as a predictor for diabetes in a metagenome wide association study (Qin et al., 2012), a disease exhibiting a higher risk for severe COVID-19 illness (Corrao et al., 2021). Depletion of *Faecalibacterium prausnitzii* was also associated with SARS-CoV-2 infection (Tao et al., 2020) or COVID-19 disease severity (Zuo et al., 2020; Yeoh et al., 2021) in studies from Asian populations. A recent study investigating the gut microbiome linked to COVID-19 severity in a cohort from Portugal also reported a reduction of *Roseburia* genus in patients with severe COVID-19 (Moreira-Rosário et al., 2021). Interestingly, SARS-CoV-2 infection reduced the short-chain fatty acid production in feces in nonhuman primate macaques and was associated with alterations of the bacterial network of the gut microbiome in these animals (Sokol et al., 2021).

Our study is a single center study from a European tertiary care hospital and revealed clear differences in the gut microbiome between SARS-CoV-2 positive and negative patients. Although SARS-CoV-2 positive and negative groups did not differ for most factors associated with alterations of the gut microbiome, we cannot exclude that other factors like diet or differences in clinical patient management, may contribute to differences observed in both groups. As sampling of the patients was performed early after hospital admission, the period for affecting the gut microbiome by clinical patient management is relatively short.

We also cannot exclude that age, comorbidities and medication contribute to differences in the gut microbiome associated with disease severity observed in our study. High age is a major risk factor for severe COVID-19, and the age of patients with severe/critical COVID-19 was significantly higher in our study compared with mild and moderate COVID-19 patients. A decrease of *Faecalibacterium* of the gut microbiome observed in severe COVID-19 of our study was also reported in studies analyzing the gut microbiome of elderly populations (Kim et al., 2019). In addition, a decrease of *Bifidobacterium*, *Bacteroides*, *Lachnospiraceae* and increase in *Akkermansia* and *Enterobacteriaceae* were among the most consistent changes of the gut microbiome reported in elderly patients (An et al., 2018; Badal et al., 2020). However, a high variation of the gut microbiome and no uniform gut microbiome signature for elderly can be inferred from the current literature (An et al., 2018). Just recently, healthy aging was associated with the compositional uniqueness of the gut microbiome and the depletion of core genera, especially *Bacteroides* (Wilmanski et al., 2021). In addition, health status, ethnicity and geographic regions have been identified as important confounding factors that may affect the gut microbiome which may be more important than age (Ghosh et al., 2020). As the depletion of SCFA-producing bacteria *Faecalibacterium* (Zuo et al., 2020; Yeoh et al., 2021) and *Roseburia* (Moreira-Rosário et al., 2021) was associated with severe COVID-19 or with SARS-CoV-2 infection (Tao et al., 2020), and experimental studies in non-human primates observed a SCFA reduction in SARS-CoV-2 infected animals, it is unlikely that age alone explains the differences observed in patients with severe COVID-19 and makes these bacteria candidates for gut microbiota manipulation to improve course of COVID-19. However, a contribution of age and comorbidities especially to the differences observed in anti-inflammatory bacterial networks in severe/critical COVID-19 cannot be excluded.

A strength of our study is the well characterization of our study population with only 3% of patients receiving antibiotic therapy at the time point of gut microbiome sampling, as antibiotics may heavily affect the gut microbiota composition. Other studies reported antibiotic usage in more than 30% of COVID-19 patients (Zuo et al., 2020; Moreira-Rosário et al., 2021; Yeoh et al., 2021; Zuo et al., 2021) and only one study reported exclusion of all patients receiving antibiotics within 4 weeks before enrolment (Gu et al., 2020b). In addition, the comparable period between first COVID-19 symptoms and rectal swab sampling indicates that differences observed between COVID-19 severity groups in our study are not explained by alterations of the gut microbiome associated with the phase of disease.

In summary, we found distinct differences in the gut microbiome in SARS-CoV-2 infected patients of a European cohort characterized by a reduced bacterial richness and a pro-inflammatory microbiome signature. In addition, patients with severe/critical COVID-19 exhibited increased pro-inflammatory immune cell markers and lower number of CD8*^+^* T cells and their gut microbiome was characterized by decreased anti-inflammatory butyrate-producing bacterial genera *Faecalibacterium* and *Roseburia*, and alterations of bacterial networks of bacteria with anti-inflammatory properties compared to patients with mild COVID-19 illness.

## Conclusions

The dysbiosis in SARS-CoV-2-infected patients with a pro-inflammatory signature of the gut microbiome and depletion of butyrate-producing anti-inflammatory bacterial genera as well as differences in bacterial networks in severe COVID-19 suggest that specific alterations of immunomodulatory bacteria contribute to the dysregulated pro-inflammatory immune response in COVID-19 patients with severe illness.

## Data Availability Statement

Sequencing data are available in the National Center for Biotechnology Information Sequence Read Archive under BioProject accession PRJNA747262.

## Ethics Statement

The studies involving human participants were reviewed and approved by Ethics Committee of the Medical Faculty of the University of Duisburg Essen (20-9237-BO). The patients/participants provided their written informed consent to participate in this study.

## Author Contributions

JKe and AW conceived the study and raised the funding. JKe, AW, JR, and OW designed the study. JR, LB, JKo, OW, SD, MK, JB, and DH collected study specimens. CF and JKe performed the experiments. JR analyzed the clinical data. JKe and FF analyzed the microbiome data. JKe and JR wrote the original manuscript and drafted it with substantial contributions from all other authors. All authors contributed to the article and approved the submitted version.

## Funding

This work was supported by the Stiftung Universitätsmedizin Essen under Grant number 20204699115.

## Conflict of Interest

The authors declare that the research was conducted in the absence of any commercial or financial relationships that could be construed as a potential conflict of interest.

## Publisher’s Note

All claims expressed in this article are solely those of the authors and do not necessarily represent those of their affiliated organizations, or those of the publisher, the editors and the reviewers. Any product that may be evaluated in this article, or claim that may be made by its manufacturer, is not guaranteed or endorsed by the publisher.

## Supplementary Material

Supplementary Figure 1
**(A)** Shannon diversity and Pielou´s evenness index in SARS-CoV-2 positive and negative patients. Kruskal-Wallis test was used to test for significant differences among groups. **(B)** Principle coordinates analysis of weighted UniFrac, unweighted UniFrac and Bray Curtis distance matrices. PERMANOVA multivariate analysis was used to test for significant differences.

Supplementary Figure 2 Different alpha diversity metrics illustrated by Shannon diversity index, observed features (ASVs) and Pielou´s evenness index in SARS-CoV-2 positive patients with (positive) and without (negative) diarrhea. **(B)** PCoA of principle coordinate 1 and 2 of weighted and unweighted UniFrac, Bray Curtis and Jaccard distance matrices in SARS-CoV-2 positive patients with (positive) and without (negative) diarrhea. **(C)** Visualization of the relative abundance of the 20 most abundant genera in SARS-CoV-2 positive patients with (positive) and without (negative) diarrhea.

Supplementary Figure 3 Illustration of total number of B cells (CD19^+^ B cells), NK cells (CD3^-^CD16^+^CD56^+^ NK cells), CD3^+^CD16^+^CD56^+^ natural-killer like T cells, HLA DR^+^ T cells and thrombocytes in COVID-19 patients according to disease severity.

## Abbreviations

ACE2, angiotensine-converting enzyme II; ASV, amplicon sequence variant; BMI, body-mass-index; CD, cluster of differentiation; COVID-19, coronavirus disease 2019; FDR, false discovery rate; HLA-DR, Human Leukocyte Antigen – DR isotype; IL, interleukin; LDA, linear discriminant analysis; LEfSe, Linear discriminant effect size analysis; OTU, operational taxonomic unit; PCoA, principle coordinates analysis; QIIME2, Quantitative Insights Into Microbial Ecology2; SARS-CoV-2, severe acute respiratory syndrome coronavirus type 2; sIL-2R, soluble IL-2 receptor; SpO2, Saturation of peripheral oxygen; WHO, World Health Organization.
