# Supplementary figures and images for: A Pro-Inflammatory Gut Microbiome Characterizes SARS-CoV-2 Infected Patients and a Reduction in the Connectivity of an Anti-Inflammatory Bacterial Network Associates With Severe COVID-19
Source: Front Cell Infect Microbiol. 2021 Nov 17;11:747816. doi: 10.3389/fcimb.2021.747816 (PMC8635721; doi:10.3389/fcimb.2021.747816)

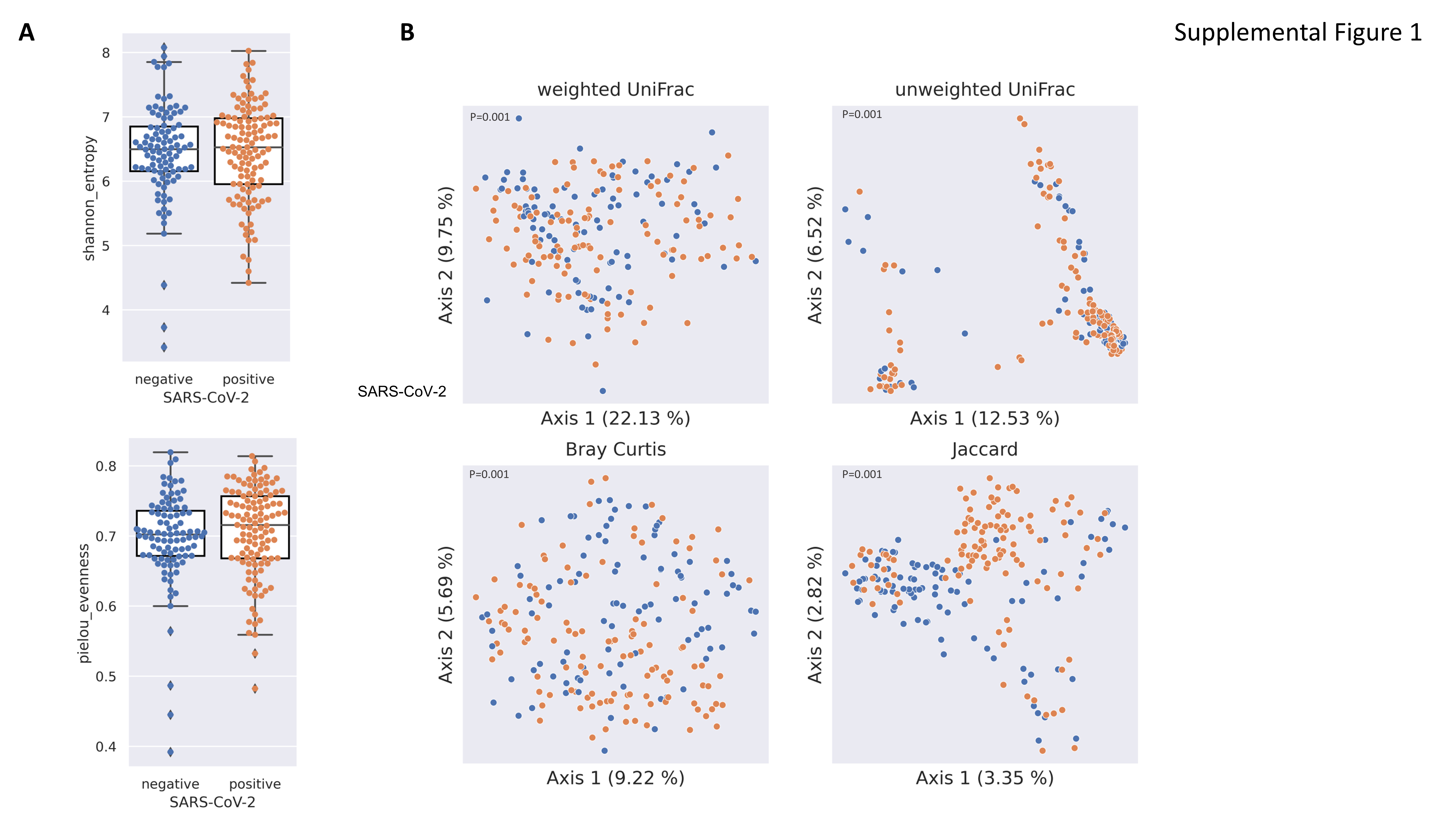

Supplement: Supplementary Figure 1 — (A) Shannon diversity and Pielou´s evenness index in SARS-CoV-2 positive and negative patients. Kruskal-Wallis test was used to test for significant differences among groups. (B) Principle coordinates analysis of weighted UniFrac, unweighted UniFrac and Bray Curtis distance matrices. PERMANOVA multivariate analysis was used to test for significant differences. [file Image_1.tif]

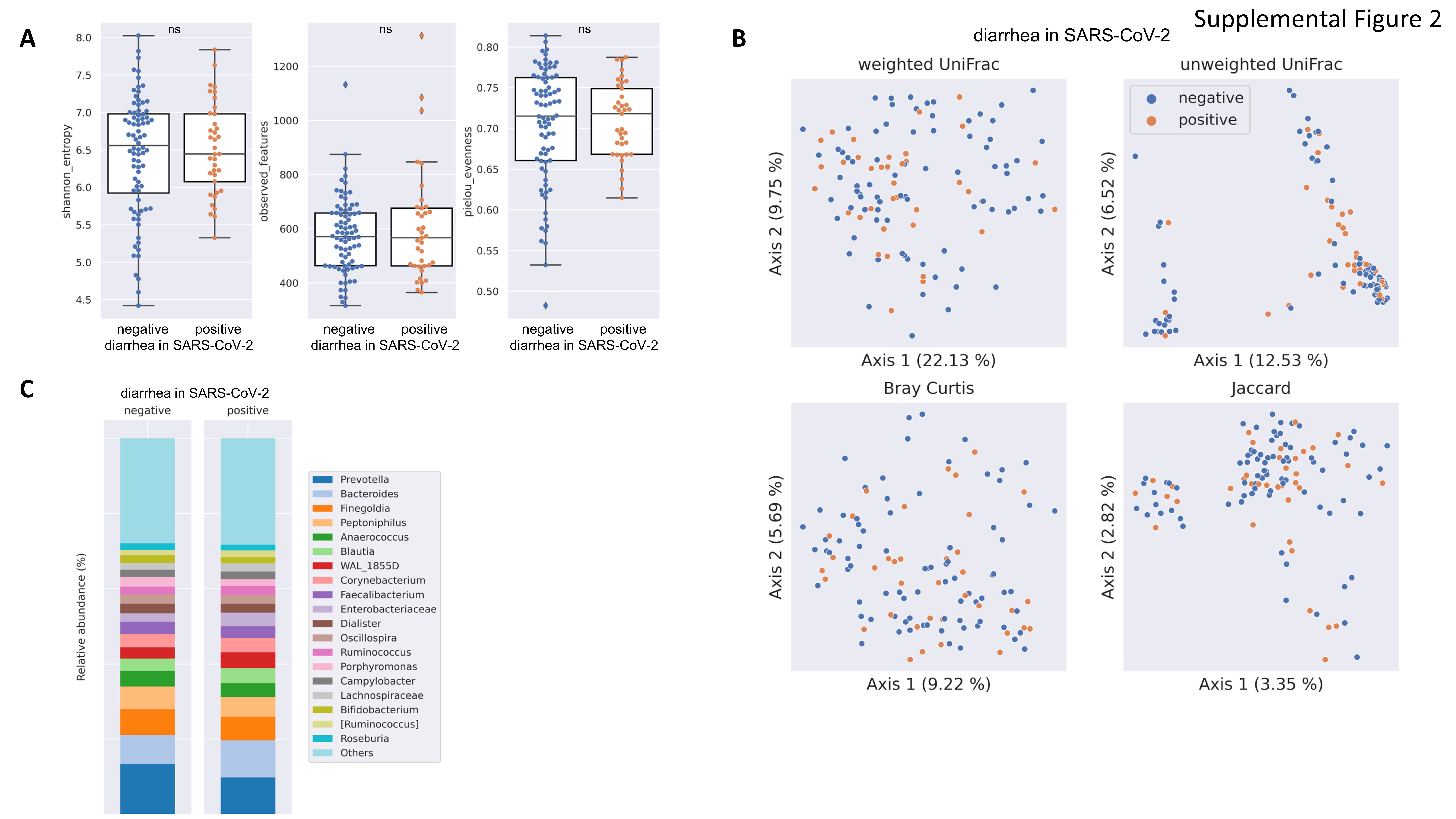

Supplement: Supplementary Figure 2 — Different alpha diversity metrics illustrated by Shannon diversity index, observed features (ASVs) and Pielou´s evenness index in SARS-CoV-2 positive patients with (positive) and without (negative) diarrhea. (B) PCoA of principle coordinate 1 and 2 of weighted and unweighted UniFrac, Bray Curtis and Jaccard distance matrices in SARS-CoV-2 positive patients with (positive) and without (negative) diarrhea. (C) Visualization of the relative abundance of the 20 most abundant genera in SARS-CoV-2 positive patients with (positive) and without (negative) diarrhea. [file Image_2.tif]

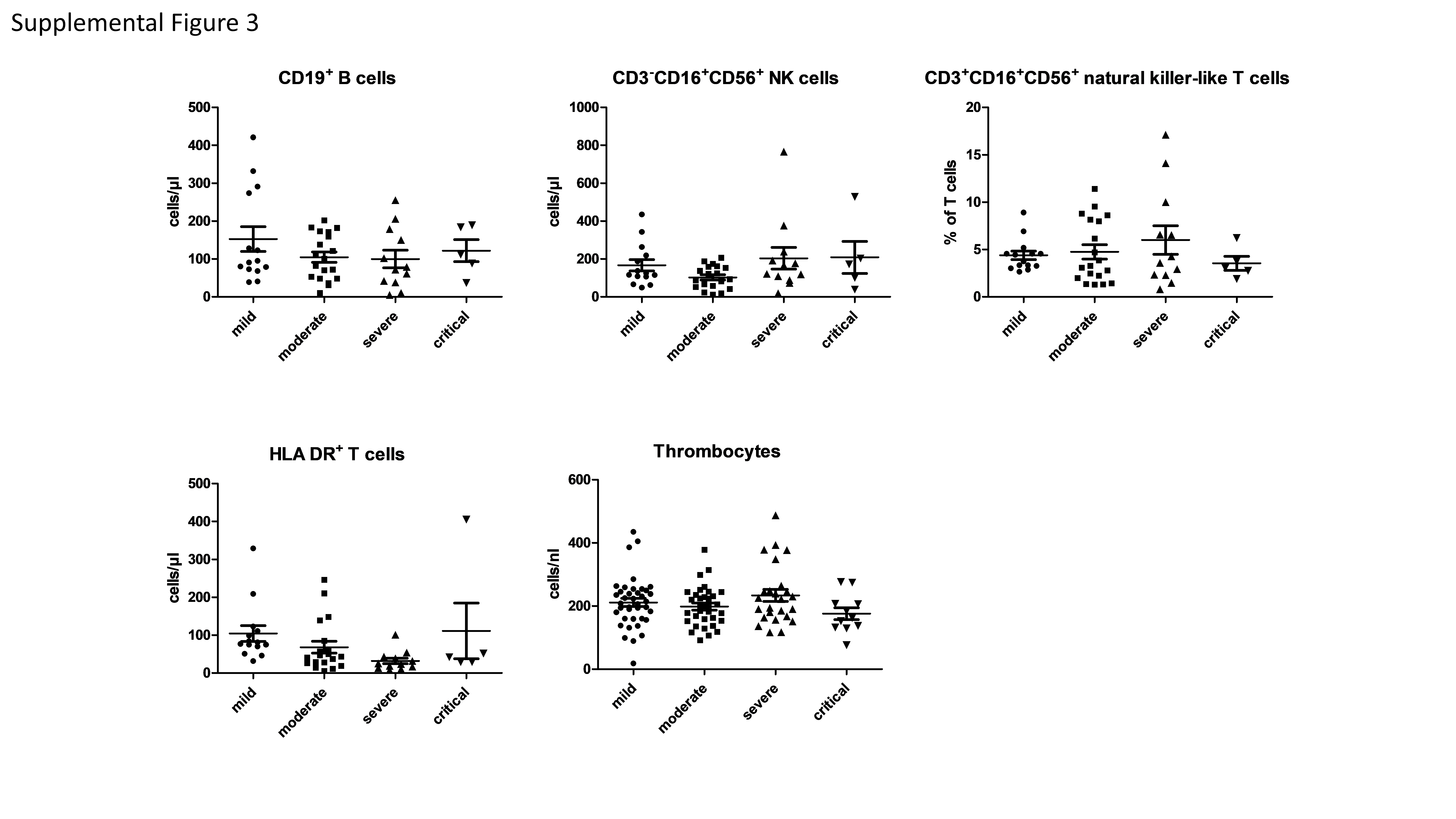

Supplement: Supplementary Figure 3 — Illustration of total number of B cells (CD19+ B cells), NK cells (CD3-CD16+CD56+ NK cells), CD3+CD16+CD56+ natural-killer like T cells, HLA DR+ T cells and thrombocytes in COVID-19 patients according to disease severity. [file Image_3.tif]
